# Emotional Labor and Occupational Well-Being: Latent Profile Transition Analysis Approach

**DOI:** 10.3389/fpsyg.2018.01084

**Published:** 2018-07-03

**Authors:** Francis Cheung, Vivian M. C. Lun, Mike W. -L. Cheung

**Affiliations:** ^1^Department of Applied Psychology, Lingnan University, Tuen Mun, Hong Kong; ^2^National University of Singapore, Singapore, Singapore

**Keywords:** emotional labor, latent profile transition analysis, job satisfaction, workload, psychological distress

## Abstract

This study used the latent profile transition analysis (LPTA) to analyze whether emotional labor profiles change across time and how these profiles relate to occupational well-being (i.e., job satisfaction, quality of work life, psychological distress, and work–family conflict). A total of 155 full-time Chinese employees completed the questionnaire survey at two time points. Three latent profiles were identified at Time 1 and the same profiles were replicated at Time 2. We determined that the majority of the participants retained the original profiles. Lastly, occupational well-being differed significantly across the identified profiles. The limitations and implications of this study were also provided.

## Introduction

Emotional labor refers to the employees’ management of emotions in accordance with organizational display rules (Hochschild, 1983; Grandey, 2003). Researchers have started to explore the association between emotional labor and occupational well-being through the person-centered approach, such as latent profile analysis (LPA) (e.g., Cheung and Lun, 2015; Cossette and Hess, 2015; Gabriel et al., 2015). Their goal is to identify whether particular subgroups exhibit certain patterns in using emotional labor strategies. The adoption of such approach is important because it provides a considerably realistic estimation of the use of emotional labor and its impact on occupational well-being. However, the existing LPA emotional labor analysis fails to consider the potential of profile shift at different time points. The current study will adopt the latent profile transition analysis (LPTA) to analyze whether emotional labor profiles change across time and how these changes relate to health and job outcomes.

### Emotional Labor: Personal-Centered Approach

Although emotional labor has been conceptualized differently in the last two decades (see Grandey et al., 2013; Grandey and Melloy, 2017 for a review), current research frequently conceptualizes such idea as a regulation process. Subsequent studies have identified three emotional labor strategies, namely, surface acting, deep acting, and the expression of naturally felt emotion. Surface acting refers to the change of emotional expression without altering the inner emotional state. A typical example is that employees simply fake a smile when they are interacting with clients. Deep acting requires the modification of employees’ inner feelings to express the desired emotion of the organization. To express positive emotion, employees may use a variety of strategies, such as diverting attention, to facilitate the change of inner emotion that is consistent with the external emotional display. Lastly, the expression of naturally felt emotion involves spontaneously experiencing and displaying the felt emotion in the workplace, that is, employees use their genuine emotion when they interact with their clients (Diefendorff et al., 2005). In general, the results support the idea that the use of different emotional labor strategies will lead to different outcomes. For example, surface acting is related to considerably low job satisfaction and heightened burnout. However, deep acting did not relate to a negative occupational well-being but was positively related to job performance (for a meta-analytic review, see Hulsheger and Schewe, 2011). Compared to surface acting and deep acting, study on the outcome of performing the expression of naturally felt emotion is relatively limited. Conservation of resource (COR) model is often used to explain why the use of different emotional labor strategies leads to different health and job outcomes. For surface acting and deep acting, both are conceptualized as effortful emotional regulation (Gross, 1998) in which employees have to invest their emotional resources to perform these strategies. Based on COR model, when employees continue to invest resources without replenishment, they will experience threatened or actual loss of valuable resources. By surface acting, employees have to constantly regulate their emotions in the workplace without resource replenishment. It often leads to higher sense of emotional dissonance, which is detrimental to employees’ psychological well-being. By deep acting, even though employees have to invest valuable resources, such regulation is often resulted in higher sense of authenticity and better job satisfaction, which can offset the net loss of resources. Finally, for the expression of naturally felt emotion, it is a form of automatic regulation process which requires minimal resource input, and employees can enjoy a higher sense of self emotional congruence because they do not need to actively regulate their emotions. Thus, it is not surprising that expression of naturally felt emotion often results in better outcomes, such as heightened job satisfaction and better psychological well-being (e.g., Cheung and Tang, 2010).

These results primarily derived from a variable-centered approach, that is, one-to-one mappings between each emotional labor strategy and occupational well-being. However, such mapping may not be generalized to real-life situations because employees are unlikely to deploy only one type of emotional labor strategy at work. For example, a service provider can choose to adopt surface acting in a few occasions while he or she can use deep acting or naturally felt emotions in other service encounters. Therefore, to completely understand how the use of emotional labor relates to occupational well-being, how individuals’ behavioral pattern (i.e., combination of emotional labor use) relates to occupational well-being should be understood rather than merely analyze the use of independent emotional labor strategy and its impact.

To address this concern, researchers have recently explored the association between emotional labor and occupational well-being using a person-centered approach, such as latent profile analysis (LPA). LPA is a multivariate statistical model, which posits that unobserved or underlying grouping variables (i.e., a latent class) can be inferred from a set of indicators. The goal is to identify whether particular subgroups exhibit certain patterns of the use of emotional labor strategies. Gabriel et al. (2015) focused on the dimensions of acting and deep acting and reported five profiles of emotional labor, namely, deep actors (employees using primarily deep acting), non-actors (employees using similar levels of the two strategies that could not be considered moderate or high), low actors (employees not using surface acting nor deep acting), regulators (employees using both of these strategies), and surface actors (employees primarily using surface acting). The results showed that surface actors tend to report lower job satisfaction but higher emotional exhaustion and felt more inauthentic than the other profiles.

Cheung and Lun (2015) identified three classes of emotional labor usage based on the analysis of the three emotional labor strategies as applied to a sample comprising Chinese teachers: active actors (the use of all three strategies), display rule compliers (represented by higher use of surface acting and deep acting), and emotionally congruent employees (represented by the high use of expression of naturally felt emotion and deep acting). The aforementioned study also compared the levels of burnout and job satisfaction among the three identified classes and determined that emotionally congruent employees tend to report substantially low burnout and high job satisfaction. Meanwhile, the display rule complier reported the highest burnout and low job satisfaction.

Cossette and Hess (2015) adopted LPA to analyze the use of emotional regulation processes in the workplace. Unlike the preceding studies, Cossette and Hess (2015) measured suppression (conceptually similar to surface acting), reappraisal (a key strategy to facilitate deep acting), and naturally felt emotions. In study 1, four emotional labor profiles were identified. These profiles include flexible (using all three strategies), authentic (primarily uses reappraisal and naturally felt emotion), suppressors (uses adopting suppression), and non-regulators (do not actively use these regulation strategies). In study 2, the original profiles were identified and two additional profiles were obtained, including actors (primarily using reappraisal and suppression) and reappraisers (primarily using reappraisal).

### Emotional Labor by LPTA

Latent profile analysis provides invaluable information on how emotional labor as profiles relate to occupational well-being. The underlying assumption of this approach is treating emotional labor as a static regulation pattern. From a different context, the current emotional labor profiles identified by LPA is assumed to be stable across time and situations. We draw from various lines of research and argue that the use of emotional labor is a dynamic process, in which the adoption of emotional labor can change over time. For example, Stone et al. (2006) determined that emotion fluctuates within work days. Positive emotion typically peaks around noon, whereas negative emotions are likely to reach its peak at mid-morning and mid-afternoon. Thus, employees may no longer need to actively regulate their emotions at noon, when positive emotion is high. At other times when negative emotions are likely to be experienced, employees may need to use considerably active emotion regulation strategies, such as deep acting and surface acting, to ensure that their emotional display is in accordance with the organization’s requirements. Similarly, the Conservation of Resource model (Hobfoll, 1989) indicates that employees should invest energy in meeting various organizational requirements (e.g., display rules). The amount of energy/resources spent in meeting such work demands represent a loss of existing valuable resources and may become a source of stress and strain (Cheung and Tang, 2007). Employees will strive to minimize resource loss and retain and protect valued resources. Surface acting and deep acting are categorized as effortful processing, whereas the expression of naturally felt emotion is regarded as automatic processing (Cheung and Tang, 2010). When employees adopt substantially effortful processing, such as surface acting and deep acting, resources will be depleted over time, thereby leading them to experience job strain (e.g., burnout and job dissatisfaction). To cope with the resource drain, employees may adopt minimal effortful processing and shift to automatic processing strategy, such as the expression of naturally felt emotion to retain their cognitive, energetic, and emotional resources. To summarize, employees are likely to change their emotional labor strategies across time, thereby possibly leading to variations in employees’ emotional labor profiles. To the best of our understanding, no study has yet to analyze such change of emotional labor profiles. To fill in this knowledge gap, we use LPTA to track changes in the profiles of emotional labor use across time.

LPTA is the extension of LPA and uses longitudinal data to identify movement between subgroups over time (Lanza et al., 2010). The focus of LPTA is on the transition probabilities between qualitatively different status or membership changes. By adopting LPTA, latent status membership probabilities (i.e., the proportion of employees expected to belong in each latent status at each time period) and transition probabilities (i.e., probability of transitioning from a particular latent status at one time to another status at another time points) will be obtained to indicate the change of profiles.

We will analyze how these profiles relate to employees’ well-being across two time points. To determine the selection of the well-being variables, we followed the recently revised emotional labor model of Grandey and Melloy (2017). In particular, the aforementioned study suggested through research the inclusion of multi-facet well-being variables. To achieve this goal, we have included health outcome (i.e., psychological distress), relational outcome (i.e., work–family conflict), and attitudinal outcomes (i.e., job satisfaction and quality of work life) as additional variables for our study.

## Materials and Methods

### Participant

This was a two-wave over time study. Employees were call-center representatives and customer service representatives in retail stores. At Time 1, participants completed a questionnaire and return it to the human resource staff in sealed envelopes. The administrative staff would then forward the completed questionnaires to the first author. In order to match the questionnaires at Time 2, which were 6 months after T1, participants provided their staff identification number and email address. 206 valid questionnaires were received at Time 1, with a return rate of approximately 95%. Among participants who reported their gender, 81 were men and 117 were women (Mean age = 31.20 years, *SD* = 10.42 years; mean tenure = 8.28 years, *SD* = 10.62 years). At Time 2, a total of 205 questionnaires were sent and 150 completed questionnaires were collected. The return rate was 76%. Among those who reported their gender, 59 were men, 90 were women, and one was unidentified.

### Attrition Analyses

Attrition analyses were conducted to evaluate if there was significant difference between those who had completed questionnaires at both Time 1 and Time 2 (completers, *n* = 155) and those who had only completed questionnaire at Time 1 (non-completers, *n* = 50) on the emotional labor strategies as well as outcomes (i.e., job satisfaction, work-to-family conflict, quality of work life, and psychological distress). Results showed that the two groups did not significantly differ on these variables at Time 1 except for deep-acting (non-completers: *M* = 3.96, *SD* = 0.57; completers: *M* = 4.30, *SD* = 0.59, *t* = -3.70, *p* < 0.01).

### Measures

#### Emotional Labor Strategies

Emotional labor strategies were measured by the emotional labor scale developed by Diefendorff et al. (2005). Seven items were used to measure surface acting, sample item includes “I put on an act in order to deal with customers in an appropriate way.” The alpha coefficients for surface acting at Time 1 and Time 2 were 0.79 and 0.85, respectively. Four items were used to measure deep acting. Sample item includes “I try to actually experience the emotion that must show to customers.” The alpha coefficients for deep acting at Time 1 and Time 2 were 0.67 and 0.74, respectively. Three items were used to measure the expression of naturally felt emotion. Sample item includes “The emotion that I show to customers comes naturally.” The alpha coefficients at Time 1 and Time 2 were 0.72 and 0.74, respectively. Participants were asked to rate the items on a 5-point Likert-scale, ranging from 1 “strongly disagree” to 5 “strongly agree.”

#### Job Satisfaction

Job satisfaction was measured with the five-item Job Satisfaction Index developed by Brayfield and Rothe (1951). Sample item includes “I feel fairly satisfied with my present job.” Participants rated their level of satisfaction from 1 “strongly disagree” to 5 “strongly agree.” The alpha coefficients were 0.75 and 0.83 for Time 1 and Time 2, respectively.

#### Work-to-Family Conflict

We used the five-item work-family conflict scale developed by Netemeyer et al. (1996) to measure perception of work-to-family conflict. Sample item includes “The demands of my work interfere with my home and family life”. Participants responded on a 7-point scale, ranging from 1 “strongly disagree” to 7 “strongly agree.” The alpha coefficients for the scale at Time 1 and Time 2 were 0.85 and 0.88, respectively.

#### Quality of Work Life

Quality of work life was assessed by the 16-item quality of work life scale (Sirgy et al., 2001). This scale measured different work-related need satisfaction, including health and safety needs, economic and family needs, social needs, and so forth. Sample item includes “I feel that my job allows me to realize my full potential.” Participants rated items on 7-point scale, ranging from 1 “very untrue” to 7 “very true.” The alpha coefficients for quality of work life at Time 1 and Time 2 were 0.89 and 0.90, respectively.

#### Psychological Distress

Psychological distress was assessed with the 12-item General Health Questionnaire (GHQ; Goldberg, 1978). Participants indicated the extent to which they experienced psychological distress, including inefficacy in handling problems and lack of self-worth. Sample item includes “Felt constantly under strain.” Participants rated these items in the past month from on a 4-point scale with 1 = “never” and 4 = “all the time.” The alpha coefficients of the scale at Time 1 and Time 2 were 0.85 and 0.85, respectively.

## Results

### Correlation Analysis

Correlation analyses were conducted to analyze the association among the major variables. Surface acting was determined to be negatively related to job satisfaction and quality of work life but positively related to psychological distress and work-to-family conflict at T1 and T2. Deep acting was positively related to quality of work life at T1 and T2 but negatively related to psychological distress at T2. Lastly, the expression of naturally felt emotion was positively related to job satisfaction and quality of work life but negatively related to psychological distress and work-to-family conflict at T1 and T2. **Table 1** presents the details of the correlation results.

**Table 1:** Descriptive Statistics.

	1	2	3	4	5	6	7	8	9	10	11	12	13	14
(1) Surface acting_T1	(0.79)													
(2) Deep acting_T1	0.21^∗^	(0.67)												
(3) Expression of naturally felt emotion_T1	-0.65^∗∗^	0.06	(0.72)											
(4) Job satisfaction_T1	-0.29^∗∗^	0.23^∗∗^	0.34^∗∗^	(0.75)										
(5) Work-to-family conflict_T1	0.38^∗∗^	-0.04	-0.32^∗∗^	-0.10	(0.85)									
(6) Quality of work life_T1	-0.31^∗∗^	0.20^∗^	0.34^∗∗^	0.58^∗∗^	-0.31^∗∗^	(0.89)								
(7) Psychological distress _T1	0.41^∗∗^	-0.09	-0.40^∗∗^	-0.40^∗∗^	0.44^∗∗^	-0.53^∗∗^	(0.85)							
(8) Surface acting_T2	0.53^∗∗^	0.01	-0.40^∗∗^	-0.18^∗^	0.16^∗^	-0.15	0.20^∗^	(0.85)						
(9) Deep acting_T2	-0.01	0.26^∗∗^	0.18^∗^	0.29^∗∗^	0.05	0.25^∗∗^	-0.12	0.02	(0.74)					
(10) Expression of naturally felt emotion_T2	-0.47^∗∗^	-0.04	0.46^∗∗^	0.29^∗∗^	-0.11	0.17^∗^	-0.26^∗∗^	-0.75^∗∗^	0.16	(0.74)				
(11) Job satisfaction_T2	-0.19^∗^	0.16^∗^	0.24^∗∗^	0.69^∗∗^	-0.05	0.51^∗∗^	-0.30^∗∗^	-0.20^∗^	0.36^∗∗^	0.31^∗∗^	(0.83)			
(12) Work-to-family conflict_T2	0.26^∗∗^	0.01	-0.34^∗∗^	-0.14	0.55^∗∗^	-0.24^∗∗^	0.31^∗∗^	0.29^∗∗^	-0.03	-0.24^∗∗^	-0.08	(0.88)		
(13) Quality of work life_T2	-0.28^∗∗^	0.10	0.25^∗∗^	0.47^∗∗^	-0.11	0.53^∗∗^	-0.30^∗∗^	-0.30^∗∗^	0.22^∗∗^	0.35^∗∗^	0.60^∗∗^	-0.28^∗∗^	(0.90)	
(14) Psychological distress_T2	0.22^∗∗^	-0.15	-0.33^∗∗^	-0.46^∗∗^	0.24^∗∗^	-0.54^∗∗^	0.64^∗∗^	0.33^∗∗^	-0.26^∗∗^	-0.37^∗∗^	-0.47^∗∗^	0.41^∗∗^	-0.56^∗∗^	(0.85)
Mean	3.13	4.30	3.35	3.73	3.35	42.48	2.05	3.06	4.03	3.28	3.54	3.58	40.39	2.07
*SD*	0.94	0.59	1.11	0.79	1.55	7.96	0.47	0.92	0.64	1.01	0.82	1.46	7.94	0.44


^∗^*p* < 0.05 and ^∗∗^*p* < 0.01.

### Latent Profile Analyses

This study began by evaluating the fit of a two-class model and systematically increased the number of latent classes in the estimation. Raw data were used as data input and maximum likelihood estimation was adopted as the estimation method. To determine model fit, we evaluated several fit indexes, including Akaike’s information criterion (AIC), Bayesian information criterion (BIC), sample-size-adjusted Bayesian information criterion (ABIC), the Lo–Mendell–Rubin (LMR LR) test, and bootstrap likelihood ratio test (BLRT) test. Although an absolute criterion for evaluating model fit has yet to be established, substantially lower AIC, BIC, and ABIC are preferred. We used the suggestion of Nylund et al. (2007) as basis to adopt the LMR LR, and BLRT tests to compare model fit between models (i.e., models with *k* and *k-*1 classes). Accordingly, a significant *p*-value would mean that the model with *k* classes fits the data better than the more parsimonious model with *k-*1 classes. With a non-significant *p*-value, the more parsimonious model is preferred. After determining the best class-solution, we used latent class membership as a between-subjective variable in the multivariate analyses of variance (MANOVA). *Post hoc* analysis with Tukey procedures will be performed thereafter to analyze the differences in covariates, including job satisfaction, quality of work life, work-to-family conflict, and psychological distress, between classes.

We first computed the latent profile models for T1. A total of five class-solutions (two-class to six-class) were computed. The measurement model failed to completely converge during the estimation except for the two-class models. This result suggests that local maxima were reached but best overall solution for the analysis was not obtained. We followed the recommendation of Jung and Wickrama (2008) and provided additional start values for the estimation. These models were re-run again but the local maxima were not reached. The best overall solution for the analysis was replicated and obtained. **Table 2** shows the fit indexes of the five tested models.

**Table 2:** Results of latent profile analyses for time 1 and time2.

Model	AIC	BIC	ABIC	Loglikelihood	LMR LR *p*-value	BLRT *p*-value	Entropy
**Time 1**							
2-class	1039.329	1078.467	1037.325	-514.480	0.081	0.040	0.714
**3-class**	**1029.515**	**1080.696**	**1026.894**	-**506.664**	**0.161**	**0.006**	**0.823**
4-class	1020.828	1084.052	1017.591	-497.758	0.043	0.020	0.859
5-class	1012.374	1087.640	1008.519	-489.809	0.498	0.510	0.847
6-class	1007.712	1095.032	1003.253	-481.187	0.026	0.011	0.872
**Time 2**							
2-class	980.612	1019.750	978.608	-489.566	0.021	0.005	0.810
**3-class**	**978.656**	**1029.837**	**976.035**	-**477.306**	**0.302**	**0.230**	**0.790**
4-class	964.895	1028.118	961.657	-472.328	0.226	0.005	0.839
5-class	957.389	1032.655	953.535	-461.447	0.054	0.010	0.866
6-class	959.943	1041.252	949.472	-456.157	0.070	0.010	0.884


The bold values indicate fit index of selected model.

Across these models, the LMR LR coefficient for the two-, three-, and five-class were not significant. For the BLRT, the coefficient for the five-class model was not significant. Both test statistics favored models with fewer classes and thus, we paid more attention to two- to four-classes models. At the second step, we analyzed the entropy, which is the summary measure for the quality of the classification in an LPA model. Values that approximate 1 indicate good classification accuracy, whereas values that approximate 0 indicate lack of accuracy. Among these models, the entropy index of the two-class model was 0.71, which was below the 0.80 convention, thereby suggesting that the classification quality was not satisfactory. Therefore, we compared the three- and four-classes models only. Results showed that the three-class model had lower BIC, whereas the four-class model had lower AIC and ABIC. Since the two models were acceptable in terms of test statistics, we then examined the characteristics of the identified profiles. We found that the three-class model exhibited clear distinction of various emotional labor profiles. However, in the four-class model, two identified classes were having very similar profile characteristics (i.e., low on surface acting but high on both deep acting and the expression of naturally felt emotion) which made the interpretation very difficult.

After considering the statistical results and interpretability of identified profiles, we had selected the three-class model at T1. First, the entropy of the three-class model was 0.823, which was above the 0.80 criterion (Ramaswamy et al., 1993; Weden and Zabin, 2005). Second, the averaged probabilities for the three-class model were 0.951, 0.928, and 0.856, which were higher than the 0.80 criterion. Third, the three-class model provides a more parsimonious model to describe the different emotional labor profiles. Theoretically, our results were also consistent with the three-class model that was previously reported (Cheung and Lun, 2015). Thus, we had adopted the three-class model at T1 based on the statistical and theoretical factors.

**FIGURE 1:**
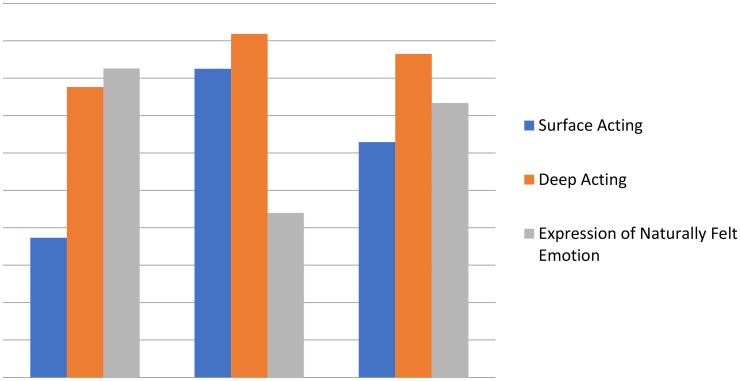
Mean scores on emotional labor items by latent class at time 1. Class 1 = Emotionally congruent employees; Class 2 = Display Rule Compliers, Class 3 = Active Actors.

The first class was labeled “emotionally congruent employees” (*n* = 34, 22.7%) because these employees adopted primarily on deep acting and the expression of naturally felt emotions. The second class was called “display rules compliers” (*n* = 43, 28.7%) because employees in this class were actively regulating their emotion based on the expected display rule via effortful processing (i.e., surface acting and deep acting). The third class was termed “active actor” (*n* = 73, 48.7%) because these employees adopted all three emotional labor strategies. **Figure 1** shows the average scores on each dimensions of emotional labor across the three latent classes.

A similar procedure was conducted for LPA at T2. A total of five models (from two-class to six-class) were computed. Similar at T1, we have provided the same start values (i.e., 400 and 100) for initial estimation. However, when estimating the 2-class model, the results suggested that local maxima were reached and the best overall solution for the analysis was not obtained. We followed the recommendation of Jung and Wickrama (2008) and re-ran the model using the new starting values of 400 and 100. The best log likelihood value has been replicated and the model estimation terminated normally. Subsequent models were using these starting values in estimation.

When analyzing the fit indices, the non-significant LMR and LR values from the three-class model onward generally suggested that the more parsimonious model was preferred. Given that the three-class model was adopted in T1, we substantially analyzed the two and three class model. When comparing measurement models, the statistical indicators and the meaning of each of the classes should be considered when interpreting the results yielded with LPA (Nylund et al., 2007). In this case, the measurement model may not make theoretical sense and should not be adopted when the classes suggested by LPA are meaningless in terms of understanding the emotional labor profiles. Moreover, how the measurement model will be used in subsequent LPTA should be considered given that the measurement model defines the outcome used for the study of change (Lanza et al., 2010). Therefore, we adopted the three-class model at T2 as well.

**FIGURE 2:**
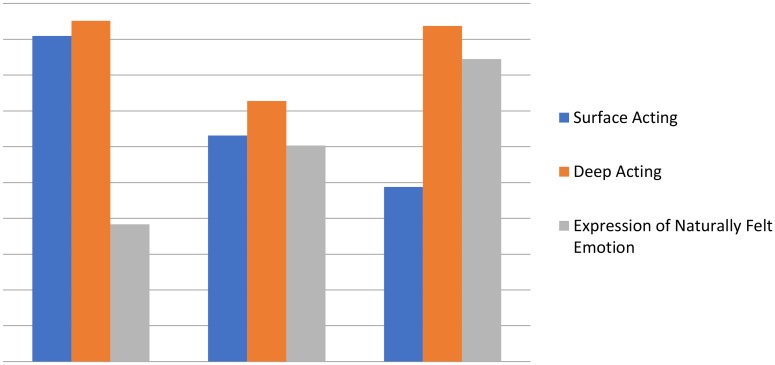
Mean scores on emotional labor items by latent class at time 2. Class 1 = Emotionally congruent employees; Class 2 = Display Rule Compliers, Class 3 = Active Actors.

Upon analysis of the average latent class probabilities in the three-class model, we determined that the probabilities were 0.957, 0.894, and 0.899, which were above the 0.80 convention. The three identified classes also corresponded well with the three classes identified at T1, namely, active actors (*n* = 91, 61%), display rule compilers (*n* = 15, 10.1%), and emotionally congruent workers (*n* = 44, 29.1%). **Figure 2** shows the average scores on each dimensions of emotional labor across the three latent classes.

### LPTA

**Table 3** presents the transition probabilities of the LPTA model. In general, the results suggest that the emotional labor profiles were fairly consistent across time, particularly for the active actors (class 1: transition probability = 0.689) and emotionally congruent employees (Class 2: transition probability = 0.678). That is, 69% of the active actors and 68% of the emotionally congruent employees at T1 remained in their respective classes at T2. For the display rule compliers (class 3), we determined an interesting transition. In particular, the probability of employees staying in the same profile was only 0.364. A large number has moved to active actor profile (class 1: transition probability = 0.475) and to a limited extent, to emotionally congruent employees (transition probability = 0.161). We determined that the “active actor” was the most popular profile at the two time points (*n* = 73 and 91 at T1 and T2, respectively) and the size of the emotionally congruent profile is relatively stable (*n* = 34 and 44 at T1 and T2, respectively). However, the size of the display rule compliers shrunk considerably across time (*n* = 43 and 15 at T1 and Time 2, respectively).

**Table 3:** Latent probabilities for the latent profile transition analysis model.

	Time 2		
	
	Active actor	Emotionally congruent employees	Display rule compliers
**Time 1**			
Active actor	0.689	0.138	0.173
Emotionally congruent employees	0.305	0.678	0.017
Display rule compliers	0.475	0.161	0.364


### Latent Profiles and Outcomes

We performed MANOVA using SPSS 22.0 on the outcome variables (i.e., job satisfaction, work-family conflict, quality of work life, and psychological distress) to explore the differences between emotional labor profiles across the two-time points. In the analyses, a significant multivariate effect (*p* < 0.05) was followed up with *post hoc* comparisons of the group means. Partial eta-squared (η^2^) and their 95% confidence intervals are used to indicate the effect size.

MANOVA was conducted separately for T1 and T2. In T1, Wilks’s Lambda = 0.81, *F* = 3.88, *p* < 0.001, η_p_^2^ = 0.10. In T2, Wilks’s Lambda = 0.79, *F* = 4.55, *p* < 0.001, η_p_^2^ = 0.11. We then performed two separate ANOVAs for outcome measures at T1 and T2 of the three identified profiles. In order to control for the inflated Type 1 errors, Bonferroni corrections were used. Across both time points, emotionally congruent employees reported the highest levels of job satisfaction and quality of work life and the lowest levels of work-to-family conflict and psychological distress. By contrast, the display rule compliers reported substantially lower levels of job satisfaction and quality of work life but high levels of psychological distress and work-to-family conflict at T1. They also had the lowest quality of work life but higher psychological distress at T2. **Tables 4, 5** present the detailed results of these analyses on the outcomes.

**Table 4:** Means comparison for covariates across latent profiles at time 1.

	1	2	3	*F*	95% CI	Partial η^2^
Job satisfaction_T1	3.78^a^	3.42^a^	3.59	2.38	3.47–3.70	0.04
Quality of work life_T1	44.61^a^	39.41^a,b^	43.33^b^	5.05^∗∗^	41.22–43.74	0.08
Psychological distress_T1	1.82^a^	2.29^a,b^	2.02^b^	10.95^∗∗^	1.98–2.12	0.13
Work-to-family conflict_T1	2.87^a^	4.17^a,b^	3.10^b^	9.45^∗∗^	3.11–3.59	0.11


*Class 1 = Emotionally congruent employees, Class 2 = Display rule compliers, Class 3 = Active actors. Cells with matching superscripts have significantly different (p < 0.05) mean values. Bonferroni correction was used to control for Type 1 error.*

**Table 5:** Means comparison for covariates across latent profiles at time 2.

	Class 1	Class 2	Class 3	*F*	95% CI	Partial η^2^
Job satisfaction_T2	3.72^a^	3.31	3.26^a^	6.46^∗∗^	3.32–3.56	0.13
Quality of work life_T2	43.31^a^	35.36^a,b^	39.54^a,b^	9.13^∗∗^	39.17–41.61	0.11
Psychological distress_T2	1.86^a^	2.24^a^	2.18^a^	11.79^∗∗^	2.00–2.13	0.10
Work-to-family conflict_T2	3.23^a^	4.26^a^	3.67	4.09^∗^	3.35–3.81	0.02


*Class 1 = Emotionally congruent employee, Class 2 = Display rule compliers; Class 3 = Active actors. Cells with matching superscripts have significantly different (p < 0.05) mean values. Bonferroni correction was used to control for Type 1 error.*

## Discussion

This study applied LPTA to analyze latent profile changes and how the membership relates to occupational well-being. The results of the LPTA models describe multifaceted behavioral profiles over time. These models also allow for the identification of the associations between various behavioral profiles and outcomes, such as considerably low occupational well-being. In general, we obtained relatively stable profiles for active actors and emotionally congruent employees. However, the profile of the display rule compliers show considerable changes as indicated by the high transition probabilities. Cheung and Tang (2010) indicated that surface acting and deep acting are considered effortful processing that require the use of resources. If such resources are not replenished over time, then employees may switch to other emotional labor strategies that require limited emotional resources input, such as the expression of naturally felt emotion (i.e., emotional congruent employees).

Our findings emphasize the necessity of reconsidering the true impact of performing emotional labor. For example, deep acting has been frequently advocated to be an improved emotional labor strategy because it was determined to associate with improved health and job outcomes, such as high job satisfaction and service quality. However, the current study’s use of LPA and LPTA enabled us to determine that high use of deep acting did not necessarily relate to improved outcomes. In particular, employees who used high deep acting in conjunction with surface acting (i.e., display rule compliers) reported the lowest well-being among the three identified classes. Therefore, the suggestion of considerably using deep acting to promote well-being at work appears to be oversimplifying the situation. Our results suggest that the expression of naturally felt emotion may be the key for an improved well-being because employees using deep acting together with expression of naturally felt emotions tend to report the best outcomes. The use of the person-centered approach, such as LPA and LPTA, enable researchers to re-analyze the hypotheses formulated based on the traditional variable-centered approach, thereby eventually advancing our theories on organizational behaviors.

### Implication and Future Research

Previous studies often advocate the use of deep acting because it is related to better occupational well-being when compared to the use of surface acting. Based on our findings, we suggested that the use of deep acting does not necessarily lead to an improved occupational well-being. This result is consistent with Cheung and Lun (2015) and Gabriel et al. (2015). In particular, we determined that if employees tend to use deep acting in conjunction with the expression of naturally felt emotion, then they will generally report an improved occupational well-being. We also determined that people who express naturally felt emotion in combination with other strategies, particularly in deep acting, tend to report high job satisfaction and low burnout. Organizations may consider promoting the use of this strategy by personnel selection and establishing the necessary organizational culture.

Employees who often naturally express the organizationally desired emotions are certainly beneficial in terms of personnel selection. Individuals with several personal traits, such as extraversion and emotional expressivity, would be desirable because these traits are positively found to correlate with the expression of naturally felt emotion (Diefendorff et al., 2005). In terms of organizational culture, organizations may also grant substantial autonomy to their employees in terms of emotional expression. In particular, when organizations rigorously uphold the display rule, employees may have to express certain emotions that they do not necessarily feel.

### Limitation

This study has several limitations and results should be interpreted with caution. First, this study used a self-reported questionnaire design and thus common method variance might inflate the observed associations (Podsakoff et al., 2003). Harman’s one-factor test was conducted via exploratory factor analysis (EFA) to address the issue of common method variance. Following the procedure by Podsakoff et al. (2003), an unrotated EFA was ran with all items. If method variance is largely responsible for the covariation among the measures, the EFA should indicate that a single (method) factor account for majority of the variance. Results showed that the EFA could account for 71% of variance and the first extracted factor could only account for 22% of variance. Based on this result, common method variance has not significantly inflated the observed relations. However, we still suggest that for future studies, researchers should obtain data from other sources (e.g., supervisors and customers) for external validation. Second, our study only included employees in Hong Kong and China. Consequently, we are uncertain whether the latent profiles derived in this study is unique to the Chinese culture, particularly when previous studies suggested that the outcome of performing emotional labor may vary across cultures (e.g., Allen et al., 2014). Thus, the cross-validation of the latent profiles with a different sample is important (Wang and Hanges, 2011). Accordingly, we recommend additional research to uncover if similar latent profiles can be found in different cultural settings. The need for additional study to cross-validate the profiles identified is particularly important, given that our sample size is relatively small. At present, no clear rule has been formulated to determine the sample size for latent profile analysis. However, Tein et al. (2013) suggested that the number of profiles detected in LPA and LPTA is influenced by the sample size, with a smaller sample producing only a few profiles. Therefore, the reason for obtaining three classes in our study could be that this finding represents the true number of profiles in the population or is the result of the relatively small sample size. Although the number of profiles identified is similar to a previous study in the Chinese work context (i.e., Cheung and Lun, 2015), additional studies, preferably with a large sample size, is warranted to provide cross-validation of the profiles identified.

## Conclusion

The use of the person-centered approach is becoming considerably popular in organizational psychology (e.g., Bennett et al., 2016; Dahling et al., 2017). Our study, together with previous LPA studies (e.g., Cheung and Lun, 2015; Cossette and Hess, 2015; Gabriel et al., 2015), suggest a new approach to understand the relations between the use of emotional labor and occupational well-being. Across these studies, the findings suggest that understanding only one emotional labor strategy is insufficient. Instead, understanding how the combination of various emotional labor use relates to occupational well-being would be substantially fruitful and realistic.

## Ethics Statement

This study was carried out in accordance with the recommendations of university research guideline by sub-committee on research ethics of the research committee, Lingnan University. The protocol was approved by the sub-committee on research ethics of the research committee, Lingnan University. All subjects gave written informed consent in accordance with the Declaration of Helsinki.

## Author Contributions

FC and VL were responsible for drafting the manuscript and running part of the analyses. MC was responsible for performing the latent profile transition analysis.

## Conflict of Interest Statement

The authors declare that the research was conducted in the absence of any commercial or financial relationships that could be construed as a potential conflict of interest.
